# Practical Strategies for Integrating Ad Hoc Simulated Participants in Emergency Medicine Education: A Faculty Guide

**DOI:** 10.7759/cureus.100025

**Published:** 2025-12-24

**Authors:** Kei U Wong, Charles Lei, Sara M Hock, Tina H Chen

**Affiliations:** 1 Emergency Medicine, Division of Pediatric Emergency Medicine, Rutgers University New Jersey Medical School, Newark, USA; 2 Emergency Medicine, Hennepin County Medical Center, Minneapolis, USA; 3 Emergency Medicine, Rush University Medical Center, Chicago, USA; 4 Emergency Medicine, Saint Louis University School of Medicine, St. Louis, USA

**Keywords:** ad hoc sp, emergency medicine education, simulated participants, simulated patients, simulated persons, simulation-based curriculum, simulation in medical education, standardized patients, teaching by simulation, teaching in emergency medicine

## Abstract

Standardized patients, simulated participants, and simulated patients (SPs) are essential in emergency medicine (EM) training, offering both structured and psychologically safe opportunities for learners to develop communication and interpersonal skills. With upcoming changes to the American Board of Emergency Medicine Certifying Exam, the importance of SP-based education in residency training has further increased. Yet many programs face various constraints that preclude consistent access to professionally trained SPs, including financial, scheduling, availability, or other resource limitations. As a result, many EM educators are increasingly turning to ad hoc SPs, here defined as individuals recruited from local networks who have not undergone formal SP training. This paper draws on the collective experience of EM educators who have worked extensively with both professionally trained and ad hoc SPs across undergraduate, graduate, and continuing medical education settings, as well as best practices recommended by the Association of Standardized Patient Educators. We outline practical recommendations for recruiting, preparing, deploying, and supporting ad hoc SPs for programs with limited access to professionally trained SPs.

## Introduction

Standardized patients, simulated participants, and simulated patients (SPs) are widely used across medical education to provide learners with safe, structured opportunities to develop and refine their clinical skills [1,2]. Although definitions vary across the literature [3], the Association of SP Educators (ASPE) distinguishes these terms based on their educational applications. Standardized patients deliver highly reproducible performances for assessment contexts; simulated patients provide more flexible, learner-responsive portrayals for formative learning; and simulated participants serve as an umbrella category that encompasses any human role player in any educational context [1].

In graduate medical education (GME), SP-based education spans a wide range of applications, from building communication and interpersonal skills [2,4-6] to high-stakes Objective Structured Clinical Examinations that evaluate clinical competence and prepare them for high-acuity scenarios unique to EM [7-9]. SPs play a critical role in emergency medicine (EM) education. Upcoming changes to the American Board of Emergency Medicine (ABEM) Certifying Exam [10] further highlight the importance of high-quality SP-based education in emergency medicine (EM) residency training. For example, new exam components will include SP-driven scenarios focusing on high-stakes conversations, such as breaking bad news or managing conflict, and assessing patient-centered communication and professionalism skills [10]. This shift underscores the need for EM residency programs to integrate robust SP-based training to prepare residents for these real-world performance evaluations.

Despite the benefits of SP-based education, many EM residency programs lack access to professionally trained SPs. The expense of maintaining a professional SP program, which includes costs for recruitment, training, quality monitoring, compensation, and overall infrastructure, is prohibitive for many institutions [11]. Geographic barriers further limit access [12], particularly in rural or underserved communities. Compensation for standardized patients (SPs) varies by institution and case complexity. Publicly available data from Clinical Skills Centers indicate hourly rates generally range from $17.50 to $26.00, providing a practical benchmark for budgeting SP-based educational activities [13-18]. As a result, in our experience, many EM programs rely on ad hoc SPs, which we define as individuals recruited by educators from their personal or professional networks who receive brief, targeted coaching for defined instances of SP-based education, often on a volunteer basis.

This paper offers a series of practical strategies for effectively integrating ad hoc SPs in contexts where professional SPs are not readily accessible.

## Technical report

Professionally trained SPs are extensively prepared in case portrayal, feedback delivery, and learner engagement, requiring high levels of support to deliver highly consistent education [1,19]. In contrast, ad hoc SPs do not receive this degree of training and cannot offer the same degree of standardization. Nonetheless, ad hoc SPs offer a range of benefits to EM residency programs if educators can anticipate and address inherent limitations (Table 1).

**Table 1:** Attributes of Professionally Trained Versus Ad Hoc SPs

	Professionally Trained SP	Ad Hoc SP
Definition	An individual meticulously trained for realistic patient portrayal, such that they are indistinguishable from a real patient.	An individual recruited on an as-needed basis for a defined instance of SP-based education. Ad hoc: formed or used for specific or immediate problems or needs; fashioned from whatever is immediately available.
Recruitment Strategy	Dedicated staff with SP expertise, such as in a clinical skills center affiliated with a medical school.	Educators’ personal and professional networks. Potential recruitment avenues include medical colleagues (physicians, nurses, pharmacists), trainees (residents, students), family, friends, and other community networks.
Training Process	Extensive, structured training by a standardized patient educator for varied educational and assessment goals, including history and consultation, communication, physical examination, feedback, and evaluation.	Brief, informal training, often from a single educator, often drawing on the ad hoc SP’s perspectives and experiences.
Cost	More expensive.	Usually volunteer, though a stipend may be offered depending on educator resources.
Advantages	Standardization of educational experience, reliable learner assessment, ability to deliver sensitive or emotionally fraught patient portrayals.	Cost-effectiveness, community involvement in learner education, opportunity to recruit ad hoc SP with direct personal experience or perspective on educational topic.
Disadvantages	Depending on the institution, common challenges include: high costs, inflexible scheduling, low prioritization of GME SP education. Some institutions may not have SP training programs altogether, making professionally trained SPs completely inaccessible.	Inconsistent patient portrayal, limited learner feedback, increased need for direct supervision and management, increased need for coaching on boundaries and role separation. Ad hoc SPs are not a replacement for comprehensive professional SP programs.

For EM programs working with ad hoc SPs, we offer a set of experience-based recommendations that account for the unique strengths and challenges of this SP population, as well as practical considerations that arise when EM faculty are responsible for training. Our recommendations are summarized as a series of key steps in the development and deployment of EM education employing ad hoc SPs (Table 2).

**Table 2:** Key Steps in Working With Ad Hoc SPs

Stage	Timeline	Key Activities
Design and Align	Months Before	Develop curriculum in alignment with educational goals. Consider the type of ad-hoc SP needed to meet patient portrayal, educational, and assessment goals. Consider financial compensation.
Recruit and Screen	Months Before	Recruit from educational or personal community, with attention to the psychological safety of the ad hoc SP. Screen for potentially sensitive topics through substantial and detailed discussion of the ask, including timeline, goals, setup, and content.
Prepare and Practice	Days to Weeks Before	Rehearse, preferably in-situ, replicating the expected emotional content and pacing. Revise setting and curriculum as needed to maintain alignment.
Deploy and Debrief	Day of Education	Reiterate safety measures, such as a safe word for exiting the educational session. Dedicate attention to the ad hoc SP with time for check-ins, de-roling, debriefing, and end-of-session feedback.
Revise and Maintain	Days to Weeks After	Further revise setting and curriculum as needed. Thank the ad hoc SP for their contribution.

Design and align

EM residency programs may have limited access to a robust pool of SPs, particularly when compared to clinical skills centers that may employ dedicated staff for SP recruitment and training. As a result, once the simulation curriculum is developed, we recommend designing a simulation scenario around the unique strengths and experiences of the ad hoc SP recruited. For example, a palliative care advocate may effectively portray a family member in an end-of-life discussion, a customer service representative may be well-suited for a de-escalation scenario, and an experienced nurse may be an effective ad hoc SP in an interprofessional communication case. This approach requires flexibility on the part of faculty, as ad hoc SPs may have lived experiences, professional backgrounds, or communication styles that do not align perfectly with the originally intended scenario design. Instead, faculty should seek to identify areas of overlap between the ad hoc SP’s skill set and the educational objectives of the session, adjusting the case accordingly to maintain educational value.

Professionally trained SPs typically memorize detailed scripts, such as the ASPE Case Development Template [20], to ensure consistent, reproducible portrayals across educational sessions. When working with ad hoc SPs, EM faculty should recognize and accept that patient portrayals will have variance. In these settings, a more concise and focused script may be appropriate, offering a limited but purposeful set of prompts and emotional cues that support the core learning objectives of the scenario. Additionally, maintaining real-time communication with the SP, such as through a radio, provides flexibility to redirect the interaction toward intended objectives. This pragmatic approach can maintain scenario fidelity while accommodating the practical limitations of working with ad hoc SPs. A variety of sample SP scripts are publicly available through educational resources such as MedEdPORTAL [21-23] and the Journal of Education and Teaching in Emergency Medicine [24,25].

Recruit and screen

Ad hoc SP recruitment often relies on the reach of the EM program’s network. When feasible, it is ideal to recruit SPs who are unknown to resident learners, as unfamiliarity supports suspension of disbelief and promotes authentic engagement in the simulation scenario. Another successful recruitment strategy is arranging interdepartmental or interinstitutional faculty exchanges, which can expand the pool of qualified SPs while preserving authenticity for learners. For example, leveraging a trauma attending as an SP for a trauma case ensures a highly realistic experience for learners.

During the recruitment process, EM faculty should clearly communicate their ask to the ad hoc SP. This includes outlining the case’s educational goals, the anticipated commitment, inclusive of preparation and deployment, the overall session format and flow, and the SP’s specific role. Without this guidance, ad hoc SPs, especially those unfamiliar with medical education, may find the pace and intensity of residency education confusing or overwhelming. Providing clear expectations, rehearsal opportunities, and access to resources such as ASPE guidelines can help mitigate these challenges and promote psychological safety.

While EM clinicians are accustomed to emotionally intense scenarios, ad hoc SPs may lack this experience. There is strong evidence that repeated exposure to distressing clinical situations can negatively impact physicians’ psychological and physical well-being [26], and the same is true for SPs in emotionally challenging roles [27,28]. Faculty should therefore review case content in advance with potential SPs and clearly describe any emotionally charged or complex elements. Ad hoc SPs should be explicitly informed that they may pause or withdraw from participation at any time, and their comfort and well-being will be prioritized throughout the simulation. For scenarios that are expected to be particularly charged, such as sexual assault, late pregnancy loss, or pediatric death disclosure, our recommended best practice is to recruit and hire a professionally trained SP for the role. If there is no opportunity to work with a professionally trained SP, an alternative is to present these emotional discussions as case studies and offer residents opportunities to practice eliciting important historical aspects in teams.

Ad hoc SPs may have personal experiences with the healthcare system, such as prior trauma, illness, pregnancy, or negative encounters with providers. While enthusiasm for participation is valuable, it does not preclude the possibility of retraumatization [29]. Faculty should engage in open dialogue with the SP about case content, encourage questions and concerns, and remain receptive to feedback. In some instances, it may be appropriate to revise aspects of the case to avoid content that closely mirrors the SP’s lived experience, thereby minimizing the risk of psychological harm or inadvertent disclosure of highly personal information.

Confidentiality is particularly important when ad hoc SPs are recruited from within professional networks. EM-interested medical students may rely on faculty for mentorship and future letters of recommendation. Clinical faculty, staff, and non-EM residents may continue to interface with the EM program in professional contexts. SP-based education can sometimes lead to the disclosure of information that would not typically occur in the work environment. EM faculty should pre-brief EM residents to reinforce the importance of maintaining confidentiality and separating the simulated experience from ongoing professional relationships. This helps preserve psychological safety for all participants and upholds professional boundaries within the educational setting.

Prepare and practice

To support a successful educational experience, EM faculty should conduct a preparatory rehearsal with the ad hoc SP prior to the scheduled simulation session. Ideally performed in situ, this rehearsal should closely replicate the timing, pacing, and content of the planned scenario. This allows the SP to become familiar with the physical space, equipment, and expectations of the session. It also provides a valuable opportunity for faculty to offer coaching, answer questions, assess the emotional demands of the case, and make any necessary modifications to improve realism, consistency, and psychological safety. Rehearsal sessions should include opportunities for SPs to provide feedback, which faculty should evaluate and apply to optimize scenario design.

While preparatory sessions can significantly enhance case fidelity and SP confidence, they often present logistical challenges due to conflicting schedules. These sessions may also be impractical for certain participants, such as pediatric SPs.

To recognize the significant time and effort of ad hoc SPs, EM faculty should consider offering small honoraria, gift cards, parking vouchers, refreshments, or other tokens of appreciation as gestures of gratitude and to promote continued engagement. Programs that require SPs regularly or for many training dates might consider larger stipends for more significant time commitments.

Deploy and debrief

EM residency educational sessions are often fast-paced, with limited time between activities. When incorporating ad hoc SPs, EM faculty should intentionally schedule regular check-ins and provide adequate break time between patient portrayals to assess SP well-being and prevent fatigue. Additionally, time should be reserved for de-roling, a structured transition that helps SPs separate themselves from the emotional and psychological aspects of the character they portrayed [29]. This process helps to protect SP's psychological safety and promote long-term engagement.

A dedicated post-session debriefing with the ad hoc SP is a critical component of the educational process. Beyond offering a chance to express appreciation in person, the debriefing allows SPs to share their unique perspectives on scenario design, logistics, and learner interactions. As the field increasingly recognizes the value of SP input in the co-creation of SP-based education [30], ad hoc SPs can contribute meaningful feedback and enhance future sessions. While ad hoc SPs may lack formal feedback training, their lived experience offers valuable insights, particularly for cases prioritizing authenticity and patient-centered communication. Decisions about including SPs in learner debriefs should align with case complexity and objectives. Importantly, SPs provide meaningful contributions throughout the process, from pre-design and delivery to post-session refinement based on observed challenges. Faculty should invite discussion about the recruitment and training process, educational day scheduling and logistics, clarity of the case script, and any unexpected challenges that arise. These conversations not only inform curriculum improvement but also foster a culture of reflection, collaboration, and respect.

Revise and maintain

After an educational session, faculty should follow up with the ad hoc SP. This is particularly important when working with individuals drawn from the EM program’s personal and professional networks. A positive working experience can encourage future participation, helping build a reliable pool of SPs for upcoming sessions. Conversely, a confusing or negative interaction may require thoughtful communication to preserve the relationship and maintain trust.

Faculty should also take the time to reflect on the session while their memory of the educational session is fresh. Gathering impressions from the SP, facilitators, and learners can help to identify opportunities to refine case content, improve logistics, and enhance the overall learning experience. Feedback from ad hoc SPs can be particularly valuable, offering a unique perspective on the realism, emotional tone, or clarity of the scenario. These insights should inform future curriculum development and guide improvements to the program’s approach to recruiting and supporting ad hoc SPs.

## Discussion

Emergency medicine (EM) programs working with ad hoc SPs face unique challenges and opportunities. This paper offers a series of practical strategies for effectively integrating ad hoc SPs into EM residency training, ensuring educational quality while navigating resource constraints. The strategies outlined in this guide emphasize adaptability and faculty engagement rather than rigid protocols. While not exhaustive, these strategies are rooted in the collective expertise of educators with experience across the continuum of simulation-based medical education. The recommendations are organized into a series of key steps for developing and implementing EM educational activities with ad hoc SPs (Table 2). 

Ad hoc SPs represent a practical solution for emergency medicine (EM) programs facing barriers to accessing professionally trained SPs [11,12,19]. While these individuals cannot provide the same level of standardization or assessment reliability as professionally trained SP [1], ad hoc SP offers unique benefits, including cost-effectiveness, flexibility, and the ability to incorporate diverse perspectives into simulation-based education. Our recommendation is to design scenarios around SP strengths, implement structured screening and preparation, prioritize psychological safety, and engage SPs in debriefing to address common challenges and promote educational quality.

This paper did not compare outcomes across simulation types, as the focus was on practical strategies for using ad hoc SPs. Future research should investigate how EM residency programs currently utilize SPs, identify barriers to equitable access, and assess the effectiveness of various SP models in simulation-based learning and assessment. Additionally, efforts should focus on developing standardized resources and tools to support the ad hoc SP community, including developing brief online training resources, to reduce variability and support psychological safety. As the field continues to evolve, particularly in response to changes in the ABEM Certifying Exam, collaborative inquiry and shared experience will be essential to ensuring high-quality, inclusive, and adaptable simulation practices across diverse training environments. For example, collaboration with drama students or semi-professional actors, particularly through partnerships with arts or theater faculty, could serve as a cost-effective strategy to enhance authenticity in low-resource settings and further exploration warrants. We hope our guidance provides a useful starting point for programs navigating the complexities of SP-based education without access to professional SPs.

## Conclusions

SPs are a powerful educational resource, providing EM residents with opportunities to develop critical skills in a safe, structured environment. While we recommend the use of professional SPs whenever possible, we acknowledge that many EM programs face significant financial and logistical barriers to accessing these highly trained individuals. In such settings, ad hoc SPs can serve as an effective and pragmatic alternative when thoughtfully deployed. Importantly, these practices not only support learner development but also promote SP well-being by upholding the ethical and professional standards central to simulation-based education. As simulation continues to adapt to evolving certification requirements and educational priorities, practical guidance such as this framework will be essential to ensuring equitable access to high-quality training across diverse institutions.
